# A survey of the current practice of the informed consent process in general surgery in the Netherlands

**DOI:** 10.1186/1754-9493-7-4

**Published:** 2013-01-21

**Authors:** Wouter KG Leclercq, Bram J Keulers, Saskia Houterman, Margot Veerman, Johan Legemaate, Marc R Scheltinga

**Affiliations:** 1 Department of Surgery, Máxima Medical Centre, de run 4600, Veldhoven 5504 DB, the Netherlands; 2 Department of Plastic Surgery, Bernhoven Hospital, Burgemeester de Kuijperlaan 7, Veghel 5461 AA, the Netherlands; 3 M.M.C. Academy, Máxima Medical Centre, de Run 4600, Veldhoven 5504 DB, the Netherlands; 4 Department of Surgery, Canisius-Wilhelmina Hospital, Weg door Jonkerbos 100, Nijmegen 6532 SZ, the Netherlands; 5 Department of Public Health, Academic Medical Center, University of Amsterdam, Meibergdreef 9, Amsterdam 1105 AZ, the Netherlands

**Keywords:** Informed consent, Surgery, Patient education, Questionnaire, Interactive tools, Training

## Abstract

**Background:**

A properly conducted surgical informed consent process (SIC) allows patients to authorize an invasive procedure with full comprehension of relevant information including involved risks. Current practice of SIC may differ from the ideal situation. The aim of this study is to evaluate whether SIC practiced by Dutch general surgeons and residents is adequate with involvement of all required elements.

**Methods:**

All members of the Dutch Society of Surgery received an online multiple choice questionnaire evaluating various aspects of SIC.

**Results:**

A total of 453 questionnaires obtained from surgeons and residents representing >95% of all Dutch hospitals were eligible for analysis (response rate 30%). Knowledge on SIC was limited as only 55% was familiar with all three basic elements (‘assessment of preconditions’, ‘provision of information’ and ‘stage of consent’). Residents performance was inferior compared to surgeons regarding most aspects of daily practice of SIC. One in 6 surgeons (17%) had faced a SIC-related complaint in the previous five years possibly illustrating suboptimal SIC implementation in daily surgical practice.

**Conclusions:**

The quality of the current SIC process is far from optimal in the Netherlands. Surgical residents require training aimed at improving awareness and skills. The SIC process is ideally supported using modern tools including web-based interactive programs. Improvement of the SIC process may enhance patient satisfaction and may possibly reduce the number of complaints.

## Dutch abstract

Please see Additional file 1 for the Dutch abstract.

## Background

**Figure 1:**
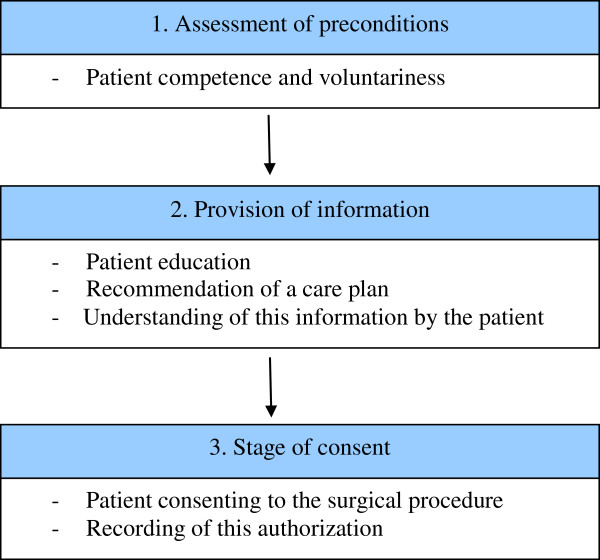
Three elements of informed consent.

It is common surgical practice to provide patients with the opportunity to consent to an operative procedure. Moreover, a surgical informed consent process (SIC) is considered a conditional element of standard surgical patient care. The basic elements presupposed by the Nuremberg and Helsinki code, the U.S. National Institute of Health and the World Health Organization are ‘assessment of preconditions’, ‘provision of information’ and the ‘stage of consent’ (Figure 1) [1-4]. A properly conducted SIC process is an interactive and structured process resulting in fully informed patients who are truly able to make an informed decision on risks and benefits of a treatment, alternative treatment options or postponing surgery [1,5-7]. Past, present and future aspects of SIC were discussed in a previous contribution [1]. Conversely, an inadequate SIC compromises patient autonomy, creates potential risks, diminishes patient satisfaction and trust in their surgeon and thereby jeopardizes the patient-physician relationship. Moreover, SIC violation may lead to a disciplinary tribunal, assault or battery action [8,9].

In the Netherlands, surgical patients are predominantly treated in 94 public hospitals around the country (8 university, 26 large teaching and 60 general hospitals). A limited number of private clinics (10%) provide a restricted volume of outpatient surgical care. All doctors including senior and junior staff are legally allowed to execute SIC. Specifics of SIC are anchored in the 1995 Dutch Medical Treatment Contract Act (Wet Geneeskundige Behandelings Overeenkomst, WGBO). More recently, the Royal Dutch Medical Association (Koninklijke Nederlandse Maatschappij ter bevordering van de Geneeskunst, KNMG) published a specific guideline in a ‘Handbook of Informed Consent’ [10]. In 2009 a guideline for ‘the preoperative route’ initiated by the Dutch Institute for Healthcare Improvement (Centraal Beleids Orgaan, CBO) was published with even more strict guidelines [11]. In 2010, the Dutch Society of Surgery (Nederlandse Vereniging voor Heelkunde, NVvH) and the Dutch Society of Anaesthesia (Nederlandse Vereniging voor Anesthesie, NVA) implemented this guideline as the gold standard [12]. Although written consent is strongly recommended, a signature of the patient or medical representative is not legally required [12].

Even though improvements in SIC were attained in recent years, other studies suggest that the implementation of SIC is still suboptimal in surgical practice [1,3]. Surgical staff may overestimate patient’s competence to make well informed decisions whereas patients are often unaware or misinformed on the role of SIC [1,3,13,14]. Surgeons may also underestimate the extent of information that is expected by their patients [15]. Some aspects of the information may not be understood. Moreover, patients understanding is not routinely checked [3,5,16-20]. Organisation and recording of the SIC process is often poor [21-23]. Training of residents may vary as considerable geographical differences in knowledge were found [14,23]. Whether these shortcomings are also present in the Netherlands is unknown.

The aim of this study is to evaluate the reported daily practice of Dutch surgeons and residents regarding characteristics of the SIC process. We hypothesized that levels of knowledge and skills are suboptimal and may differ between Dutch surgeons and residents.

## Methods

### Questionnaire

The study was initiated by the Department of Surgery of Máxima Medical Centre (MMC) in Veldhoven, the Netherlands. MMC is a teaching hospital serving a population of 350,000 patients in a semi-rural area in the south-eastern part of the country. Ethical approval was obtained from the hospital’s Institutional Review Board. A primordial version of a self designed questionnaire was tested on a limited number of staff members and residents (n=3). Criticisms and suggestions led to an improved version that was again tested on a different portion of the surgical senior and junior staff (n=5) as an online pilot questionnaire. After a final check for ambiguities and linguistic errors, the on-line questionnaire contained 23 multiple choice questions concerning general characteristics of the respondent, knowledge of SIC and various other aspects of daily practice on SIC (Additional file 2).

Endorsed and facilitated by the Dutch Society of Surgery, an e-mail linked to the online questionnaire was sent to all actively practicing surgeons and surgical residents throughout the Netherlands in the month of May 2010. This population consisted of 1,065 surgeons (90% practicing, 10% retired) and 453 other members practising in all Dutch public hospitals and private clinics (90% residents, 10% researchers, other MD’s interested in surgery). Surgeons (S) and residents (R) were the focus of the current study as both groups are regularly involved in the SIC process [23,24]. A reminder was sent to all non-responders one month later, shortly followed by an official study closure one month thereafter.

### Statistical analysis

All data were collected in an online database, checked for duplicates and immediately rendered anonymous. Statistical analyses were performed using version 18 SPSS, Chicago, Illinois, USA. Descriptive statistics were used to analyse the data. χ ^2^ and Fisher’s Exact tests were used to compare data obtained from surgeons with data from residents. A p<0.05 was considered significant.

## Results

### Representativeness

**Figure 2:**
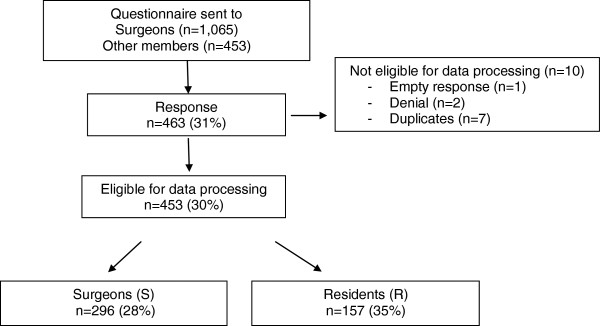
Response to surgical informed consent questionnaire.

Questionnaires were received from 96% (90/94) of all public hospitals and from four private ones. Incomplete or duplicate questionnaires were excluded from the study as well as questionnaires from researchers and other members (n=10). A total of 453 questionnaires were eligible for processing, representing a 30% response rate (453/1518; 296 surgeons (S), 157 residents (R, Figure 2). Almost one third (32%) of the respondents were experienced surgeons (>10 years), one third (33%) was 0–10 years active as a surgeon and the remaining 35% consisted of residents. Distribution over type of hospital was almost even (university 29%, teaching 36%, general 35%).

### Knowledge of SIC Elements

SIC consists of three elements (Figure 1). When asked to point these out, almost all respondents (92%) were familiar with the element of ‘provision of information’ (Table 1). However, ‘assessment of preconditions’ and ‘stage of consent’ were less known by surgeons compared to residents (competence: S 55% vs. R 73%, p<0.001; consent: S 86% vs. R 92%, p=0.041; Table 1). One-third of both groups were wrongly convinced that a signature of either surgeon or patient was necessary for a legally sound consent procedure.

**Table 1:** Which elements form an informed consent process

**Q-Nr**	**Element of SIC**	**Question: Is the following item required in SIC**	**Total Y/N % (n=453)**	**Surgeons Y/N % (n=296)**	**Residents Y/N % (n=157)**	**p-value**
18	Assessment of preconditions	Evaluation of competence	**62**/**38**%	55/45%	73/27%	<**0**.**001**
	Provision of information	Patient education	**92**/**8**%	91/9%	94/6%	0.37
	Stage of consent	Recording of consent	**88**/**12**%	86/14%	92/8%	**0**.**041**
		Patients’ signature	**31**/**69**%	29/71%	34/66%	0.30
		Surgeons’ signature	**36**/**64**%	34/66%	41/59%	0.16

Q-NR= Question number as reported in Additional file 2.

*SIC* = Surgical informed consent.

Y/N% = Percentage of persons in this category who answers positive / negative.

### SIC in daily practice

More than two-thirds (68%) of all respondents did not realize that they are obliged to inform their patients on SIC and patient rights (Question 14). Responses varied widely regarding who is in charge of providing patients with this information (Question 15). Some judged the surgeon responsible (S 42% vs. R 29%, P=0.01), whereas others thought the resident (S 24% vs. R 36%, P=0.004) or the nursing staff (S 9% vs. R 3%, P=0.01) were responsible. Some were convinced leaflets would suffice (S 12% vs. R 5%, P=0.01).

By Dutch law, a SIC is required for elective and emergency surgical procedures. Almost half (49%) of the respondents indeed followed this regimen, whereas the other half ignored this requirement (Table 2). The latter half obtained SIC only for elective procedures (28%) or decided whether a SIC was necessary on an individual basis (23%).

**Table 2:** Daily practice on surgical informed consent

**Q-Nr**	**Question**	**Answer**	**Total (n=453)**	**Surgeons (n=296)**	**Residents (n=157)**	**p-value**
5	For which type of surgical procedure a SIC is required?	All (Y/N %)	**49**/**51**%	46/54%	54/46%	0.19
		Elective (Y/N %)	**28**/**72**%	29/71%	27/73%	
		Depending on the surgeon (Y/N %)	**23**/**77**%	25/75%	19/81%	
7	Is there a check on patient competence?	Own clinical judgement (Y/N %)	**98**/**2**%	97/3%	100/0%	0.17
		Questionnaire (Y/N %)	**1**/**99**%	1/99%	0/100%	
		No control (Y/N %)	**1**/**99**%	2/98%	0/100%	
8	Is there an institutional standard on which information is provided?	Yes	**61**/%	62%	58%	0.44
		No	**39**%	38%	42%	
10	Do you inform a patient on:	The diagnosis and operation indication (Y/N %)	**99**/**1**%	99/1%	99/1%	0.61
		The surgical procedure (Y/N %)	**97**/**3**%	97/3%	96/3%	0.32
		Complications (Y/N %)	**99**/**1**%	99/1%	98/2%	0.70
		Alternative treatment options (Y/N %)	**86**/**14**%	89/11%	80/20%	**0**.**017**
11	How do you check if the patient understood the information?	Repeat Back (Y/N %)	**14**/**86**%	15/85%	13/87%	0.67
12	Is there an institutional standard on complication rates to be used?	Yes	**52**%	58%	40%	<**0**.**001**
		No	**48**%	42%	59%	
13	Which complication percentage do you use to inform your patient?	Rates from literature (Y/N %)	**67**/**33**%	73/27%	56/44%	<**0**.**001**
		Rates from own department (Y/N %)	**29**/**71**%	35/65%	17/83%	<**0**.**001**
		Personal rates (Y/N %)	**16**/**84**%	23/77%	3/97%	<**0**.**001**
17	Is there a check prior to the surgical procedure whether the SIC process is correctly completed?	Yes	**46**%*	48%*	44%*	0.39*
		No	**54**%*	52%*	56%*	

Q-NR = Question number as reported in Additional file 2.

Y/N% = Percentage of persons in this category who answers positive / negative.

*SIC* = Surgical informed consent.

* Surgeons n=295, Residents n=156.

### Elements of SIC

1. Assessment of preconditions

The first step in the SIC process is checking the patient’s competence on making an informed decision regarding his/her own body and whether this decision is made freely. The respondents almost always (98%) judged these issues on the basis of a personal impression. In contrast, questionnaires or other validated tools were hardly used (1%; Table 2).

2. Provision of information

The vast majority (98%) provided various specifics on diagnosis and surgical procedure. Surgeons performed better on discussing alternative treatment options compared to residents (S 89% vs. R 80%, p=0.017). Surprisingly, 39% claimed that there was no institutional standard on quality and quantity of information that was deemed necessary to communicate to a patient in the preoperative stage (Table 2).

Another important issue is the disclosure of potential risks and complications. Surgeons were more often aware of the department’s general agreement on complication rates to be used compared to residents (S 58%, R 40%, p<0.001, Table 2). Most respondents used a 1% or 5% complication incidence cut-off point for informing patients (34% or 51%, respectively, Question 13). If a complication was considered serious, respondents were more willing to discuss this untoward event with their patients (S 81%, R 74%, p=0.062, Question 13). Overall, surgeons used specific complication rates more frequently compared to residents. Sources were literature-based (S 73%, R 56%, p<0.001), department-specific (S 35%, R 17%, p<0.001) or based on individual results (S 23%, R 3%, p<0.001) (Table 2).

In case of complex interventions, the surgeon is legally expected to check whether the patient comprehended the information. The repeat back method (RB) is considered gold standard [16]. However, this method was only used by 14% of the respondents whereas the vast majority (86%) relied on less reliable methods including asking ‘if everything was understood’ or ‘are there any questions’, or judged on the basis of their own intuition (Table 2).

3. Stage of Consent

The use of IC forms is not obligatory in the Netherlands. However, a minority (26%) of the respondents used these forms whereas 65% made notes in the surgical record; 9% made no report of SIC at all (Question 6). Nearly half (46%) routinely checked if a SIC was obtained before starting a surgical procedure (Table 2).

### SIC support tools

Tools to support patient education were used more frequently by surgeons compared to residents (Table 3). Leaflets (97%) were popular in contrast to websites (S 37%, R 24%, p=0.008) or movies (S 16%, R 8%, p=0.019). Almost half of all respondents also relied on other staff members for patient information (48%). 76% were interested in using SIC software (S 79%, R 71%, p=0.038).

**Table 3:** Supporting tools in the surgical informed consent process

**Q**-**NR**	**Question**	**Answer**	**Total (n=453)**	**Surgeons (n=296)**	**Residents (n=157)**	**p**-**value**
9	Which supporting tools are you using for patient education?	Leaflets (Y/N %)	**97**/**3**%	98/2%	95/5%	0.071
		Websites / software (Y/N %)	**32**/**68**%	37/63%	24/76%	**0**.**008**
		Movies (Y/N %)	**14**/**86**%	16/84%	8/92%	**0**.**019**
		Information personnel (Y/N %)	**48**/**52**%	46/54%	50/50%	0.49
22	Are you interested in using SIC software?	Interested	**76**%	79%	71%	**0**.**038**
		Not interested	**24**%	21%	29%	

Q-NR = Question number as reported in Additional file 2.

Y/N % = Percentage of persons in this category who answers positive / negative.

*SIC*= Surgical Informed Consent.

### Medicolegal consequence of SIC

The majority of the respondents (94%) judged a proper SIC important for themselves (Question 19). Interestingly, just 73% thought that their patients also considered a sound SIC important (Question 20). Some 17% of the surgeons faced an officially filed complaint regarding improper SIC in the previous five years compared to 3% of the residents (p<0.001, Question 21). Non-university hospital surgeons were significantly more at risk for these complaints compared to university hospitals colleagues (21% vs. 7%, p=0.004).

## Discussion

The aim of this study was to investigate the daily practice of the SIC process by general surgeons and residents in the Netherlands. It was assumed that level of knowledge and skills were suboptimal. Results of the present study indeed confirmed this hypothesis. Interestingly, considerable differences between knowledge levels of surgeons and residents regarding various elements of SIC were identified. It may be assumed that lack of knowledge, training and structure in the SIC process may result in a suboptimal implementation in daily practice. Conversely, an optimized SIC process may enhance patient compliance, safety, satisfaction and trust, leading to an improved physician-patient relationship.

The present study is the first of its kind in the Netherlands. However, a lack of knowledge on most aspects of SIC is consistently found in various other studies investigating surgical staffs in Europe, USA and New Zealand [1,5,14,25-27]. Residents performed worse compared to surgeons in Ireland, Germany, UK and USA [23-25,28]. They do not feel confident due to a lack of training [5,13,23,24,26,29], and up to 60% of residents in the USA claimed that they never received any feedback on these issues during their residency [29,30]. In recent years, informed consent was topic of debate in the USA and the UK and improvements in care followed. However, this debate was not so intense in the Netherlands. Dutch surgeons judge the process of SIC important but they are faced with uncertainties in daily practice. Which aspects of SIC are obligatory and which are accessory? Residents were familiar with some elements of SIC but evidently lacked practical knowledge and practice on other aspects of SIC. The recently updated curriculum for Dutch surgical residents referred to SIC only twice and just in general terms [31]. Moreover, training for surgical residents is only starting to be implemented. It may well be that surgeons still improve their knowledge the hard way, that is through complaints and legal actions. Future surgical residents require optimized training in SIC using specific courses supported by supervision in daily practice. An option would be to incorporate an educational SIC programme in the early phase (year 1–2) of the surgical residency.

Structuring a SIC process will improve its quality, completeness and legal solidity. Moreover, it will improve patient satisfaction, safety and prevent high impact malpractice claims [1,32-35]. In recent years, preoperative safety programs (SURPASS) have structured and improved patient safety significant, but there was little interest in the aspects of SIC [33]. A standard SIC form was introduced and successfully implemented in daily practice in various countries including Australia and the UK [23]. According to the present study, the SIC process in the Netherlands is highly dependent on local and personal circumstances. Therefore this process requires standardization and implementation in preoperative safety programs. A substantial number of respondents would like to receive specific forms that are designed to guide doctors and patients through the steps of the SIC process [23].

Is proper introduction and implementation of SIC in daily practice considered a nuisance by surgical staffs? All steps of the process require substantial amounts of precious consultation time. Theoretically, modern tools including computer based techniques may be used to facilitate SIC. Computers provide structure, improve quality, diminish consultation time and stimulate patient commitment [1]. At present, surgeons and residents are not using these tools for SIC on a large scale but the majority claim an open mind regarding the use of interactive software in the future [23,24]. Unfortunately, development of these programs in Europe nowadays is at the level of small pilot studies [1]. In the US however, the iMed program is fully implemented [16,36]. Further studies are necessary to explore and introduce these web-based interactive programs on a larger scale in Europe [8].

The present study may suffer from several shortcomings, as it reports on the daily practice using multiple-choice questions. Reporting bias may therefore be of influence although completing the questionnaire was voluntary and results were made anonymous. Although a 30% response rate is comparable to results obtained from other studies, the topic of the present study and e-mailing rather than post mailing may have negatively influenced this response rate [5,28,37-42]. Selection bias may have been of influence even though the response rate of surgeons and residents (65% vs. 35%) closely reflects the present Dutch surgical population (70% vs. 30%). Moreover, a response was received from 96% of all hospitals whereas the existing types of hospitals were equally distributed [41]. We therefore feel that this study is representative for the current practice of surgeons and surgical residents in the Netherlands. However, the lack of validated questionnaires and few comparable studies render interpretation of our results somewhat hazardous. It is obvious that more studies are needed to confirm these results. Strengths of this study include the voluntary setting of this survey with a response of a substantial amount (n > 450) of individuals and the support of the Dutch Society of Surgery. The comparison between surgeons and residents incidentally, showed large differences in knowledge and practice. Interestingly, several respondents declared that the questionnaire itself was very instructive and opened discussions within their departments potentially suggesting an improved awareness and a more solid role of SIC in future surgical practice.

In conclusion, the quality of the current SIC process is suboptimal in the Netherlands. Surgical residents require training aimed at improving awareness and skills. The SIC process is ideally supported using modern tools including web-based interactive programs. Improvement of the SIC process may enhance patient satisfaction and may possibly reduce the number of complaints.

## Competing interests

B.J. Keulers is developing an evidence-based concept for web-based informed consent in the company Happy Patient b.v. This software is currently being tested in a pilot study with a grant of the National Quality Institute for Consultants (SKMS) and the Dutch Society of Plastic Surgeons (NVPC).

## Authors’ contributions

Research design: WL, BK, SH, MS. Data analyses: WL, SH, MV. Literature information: WL, BK, MV, JL, MS. Writing article: WL, MS. Revising article: WL, BK, SH, MV, JL, MS. All authors read and approved the final manuscript.

## Supplementary Material

Additional file 1 Dutch Abstract. Click here for file

Additional file 2 The questionnaire. Click here for file
