# Supplementary material for: A survey of the current practice of the informed consent process in general surgery in the Netherlands
Source: Patient Saf Surg. 2013 Jan 21;7:4. doi: 10.1186/1754-9493-7-4 (PMC3804026; doi:10.1186/1754-9493-7-4)
Supplement: Additional file 1 — Dutch Abstract. [file 1754-9493-7-4-S1.docx]

**Additional non-English language abstract (in Dutch)**

Achtergrond

Het doel van het preoperatieve informed consent proces (surgical informed consent, SIC) is om patiënten een weloverwogen en welgeïnformeerde keuze te laten maken over hun operatieve ingreep. De hedendaagse praktijk betreffende SIC staat mogelijk ver van de ideale situatie af. Doel van deze studie is om de dagelijkse praktijk van chirurgen en chirurgen in opleiding betreffende SIC te evalueren en te zien of deze voldoet aan de daarvoor gestelde eisen.

Methode

Alle chirurgen en andere leden van de Nederlandse Vereniging voor Heelkunde ontvingen een online multiple-choice vragenlijst betreffende de belangrijkste aspecten van SIC.

Resultaten

In totaal waren er 453 bruikbare reacties uit meer dan 95% van alle Nederlandse ziekenhuizen (respons 30%). De kennis over SIC blijkt zeer beperkt. Slechts 55% van de chirurgen bleek bekend met de drie basiselementen van SIC (‘beoordelen van de competentie van een patiënt’, ‘verstrekken van informatie’ and ‘adequaat vastleggen van de toestemming van de patiënt’). De dagelijkse praktijk liet behoorlijke verschillen tussen alle respondenten zien, maar chirurgen in opleiding scoorden significant slechter vergeleken met chirurgen. 17% van alle chirurgen kreeg de afgelopen vijf jaar te maken met een klacht betreffende SIC, wat zou kunnen wijzen op een suboptimale implementatie van SIC in de dagelijkse praktijk.

Conclusie

De kwaliteit van het preoperatieve informed consent proces is in Nederland verre van goed. Chirurgen in opleiding scoorden minder goed dan chirurgen en dienen beter geschoold te worden. Het hele SIC proces zou geformaliseerd moeten worden in protocollen. Moderne hulpmiddelen zoals interactieve softwareprogramma’s kunnen hierbij mogelijk helpen. Door het verbeteren van het SIC proces kan de patiëntentevredenheid verhoogd worden terwijl het aantal klachten mogelijk wordt verminderd.
