# Supplementary material for: A survey of the current practice of the informed consent process in general surgery in the Netherlands
Source: Patient Saf Surg. 2013 Jan 21;7:4. doi: 10.1186/1754-9493-7-4 (PMC3804026; doi:10.1186/1754-9493-7-4)
Supplement: Additional file 2 — The questionnaire. [file 1754-9493-7-4-S2.doc]

Appendix A. The questionnaire

1. Name
2. Type of surgical staff
3. Type of hospital
4. Does your department have a standard procedure describing the informed consent process?
5. For which surgical procedures is the informed consent process held?
6. In which way is the informed consent process recorded?
7. How do you verify if a patient is competent to make an informed consent about his / her surgical procedure?
8. Does your department have a standard, what information is being told to a patient in a pre-operative consult?
9. Are the following support tools used in informing your patients at your outpatient clinic?
10. Which of the following points are discussed with the patient in a pre-operative consult?
11. How do you verify if the patient has understood the information?
12. Which complications do you always discuss with your patient?
13. Which complication percentage do you use to inform your patient?
14. Is the patient being educated in what informed consent consists of?
15. If so, who is educating the patient?
16. Where is the informed consent form being stored?
17. Is there a check just before the surgical procedure if the informed consent process is being held correctly?
18. Of what elements consists the informed consent process according to you?
19. How important is the informed consent process in total patient care according to you?
20. How important is the informed consent process in total patient care according to the patient in your opinion?
21. Have you had any legal cases in which informed consent was part of the complaint?
22. Are you interested in software helping to inform patients about their surgical procedure and to fulfil the informed consent process?
23. Do you have any remarks?
